# Risk perception during the 2014–2015 Ebola outbreak in Sierra Leone

**DOI:** 10.1186/s12889-020-09648-8

**Published:** 2020-10-12

**Authors:** Maike Winters, Mohamed F. Jalloh, Paul Sengeh, Mohammad B. Jalloh, Zangin Zeebari, Helena Nordenstedt

**Affiliations:** 1 Department of Global Public Health, Karolinska Institutet, Tomtebodavägen 18A, 17717 Stockholm, Sweden; 2 Centers for Disease Control and Prevention, Atlanta, GA USA; 3 FOCUS1000, Freetown, Sierra Leone; 4 Jönköping International Business School, Jönköping, Sweden

**Keywords:** Risk perception, Infectious diseases, Epidemiology, Global health, Ebola, Epidemic

## Abstract

**Background:**

Perceived susceptibility to a disease threat (risk perception) can influence protective behaviour. This study aims to determine how exposure to information sources, knowledge and behaviours potentially influenced risk perceptions during the 2014–2015 Ebola Virus Disease outbreak in Sierra Leone.

**Methods:**

The study is based on three cross-sectional, national surveys (August 2014, *n* = 1413; October 2014, *n* = 2086; December 2014, *n* = 3540) that measured Ebola-related knowledge, attitudes, and practices in Sierra Leone. Data were pooled and composite variables were created for knowledge, misconceptions and three Ebola-specific behaviours. Risk perception was measured using a Likert-item and dichotomised into ‘no risk perception’ and ‘some risk perception’. Exposure to five information sources was dichotomised into a binary variable for exposed and unexposed. Multilevel logistic regression models were fitted to examine various associations.

**Results:**

Exposure to new media (e.g. internet) and community-level information sources (e.g. religious leaders) were positively associated with expressing risk perception. Ebola-specific knowledge and hand washing were positively associated with expressing risk perception (Adjusted OR [AOR] 1.4, 95% Confidence Interval [CI] 1.2–1.8 and AOR 1.4, 95% CI 1.1–1.7 respectively), whereas misconceptions and avoiding burials were negatively associated with risk perception, (AOR 0.7, 95% CI 0.6–0.8 and AOR 0.8, 95% CI 06–1.0, respectively).

**Conclusions:**

Our results illustrate the complexity of how individuals perceived their Ebola acquisition risk based on the way they received information, what they knew about Ebola, and actions they took to protect themselves. Community-level information sources may help to align the public’s perceived risk with their actual epidemiological risk. As part of global health security efforts, increased investments are needed for community-level engagements that allow for two-way communication during health emergencies.

## Background

In a small remote village in Guinea in December 2013, an 18-month-old boy suddenly became very ill and died [1]. The virus that killed him was later confirmed to be Ebola. In a few months, the outbreak spread to neighbouring Liberia and Sierra Leone [2]. The Ebola outbreak had already spread to the capital cities of all three affected countries by the time the World Health Organization declared it a Public Health Emergency of International Concern in August 2014 [2]. It took more than 2 years to stop the Ebola outbreak in West Africa. In the three most heavily affected countries, more than 28,000 people became infected, of which more than 11,000 died [3].

Fear, worry and perception of Ebola risk spread across the globe despite the majority of cases occurring in West Africa. People in countries far away from the actual epidemic reportedly felt at risk of getting Ebola [4, 5]. The few Ebola cases that occurred in Europe and the United States might have exacerbated the risk perception in places where there was virtually no transmission risk [2]. On the other hand, in Ghana, closer to the actual epicentre of the Ebola epidemic, a majority of 62% of survey respondents in the Greater Accra Region reported that they did not feel at risk of contracting Ebola [6].

In Sierra Leone, the country with the largest number of Ebola cases, risk perception was expectedly elevated, with 58% of survey respondents reporting that they felt at-risk of contracting Ebola in August 2014—just 3 months from the declaration of the country’s Ebola outbreak [7]. A different survey conducted in March 2015 in Sierra Leone, several months after the peak of the outbreak, found that 90% of respondents felt that they were at-risk of contracting Ebola [8].

Risk perception is defined as “people’s subjective judgments about the likelihood of negative occurrences such as an injury, illness, disease and death” [9]. According to the psychometric paradigm, risk perception depends on a wide range of characteristics of that risk [10]. For instance, risks that are not controllable, have catastrophic potential, are certain to be fatal, and where the effects are immediate and not known to science, are more likely to be perceived as more dangerous [10]. The Ebola outbreak ticked all of those boxes.

Worldwide fears of an Ebola pandemic were likely further fuelled by non-stop media coverage of the outbreak [11]. The media’s role in the perception of risk is highlighted in the Social Amplification of Risk Framework [12]. This framework states that social, psychological and cultural processes all have the potential to heighten or to attenuate the perceived risk. An especially important role in this framework is assigned to communication, both through interpersonal communication and through the news media. The media generally favours ‘newsworthy stories’ that are new, unusual or dramatic, thereby potentially amplifying the perceived risk [12]. A survey in Germany showed that people who increased their media use to keep informed about Ebola were more likely to feel worried about the outbreak [5]. An analysis of news coverage of the Ebola outbreak in the United States found that 96% of the analysed news stories contained at least one risk-elevating message, whereas 55% of the news stories contained at least one risk-minimizing message [11].

In a situation like the Ebola epidemic or the ongoing covid-19 pandemic, large-scale behaviour change of the public is needed to curb the outbreak [13]. In many West African communities, people needed to stop traditional burial practices such as washing and touching corpses, as this formed an important risk of Ebola infection [14]. Risk communication and social mobilization interventions aimed to inform and engage the public to elicit the desired behavioural change. Previous research in Sierra Leone found that exposure to information sources was associated with increased Ebola-specific knowledge and protective behaviours [15]. Risk perception is thought to be an important determinant of behaviour change [16, 17]. According to WHO’s risk communication guidelines, ‘risk perception is the primary predictor for disaster prevention and mitigation behaviours’ [17]. Behaviour change models such as the Health Belief Model point to the importance of risk perception in influencing behaviour change [18]. Vice versa, risk perception can in turn be influenced by behaviour change, as put forward in the risk reappraisal hypothesis [19].

Not much is known about the determinants of risk perception and how knowledge and behaviours influence risk perception. Therefore, this study aims to investigate the association between exposure to information sources and risk perception in the Ebola outbreak in Sierra Leone. Furthermore, it investigates how behaviour and knowledge may be associated with risk perception.

## Methods

Three Knowledge, Attitudes and Practices (KAP) surveys were carried out in 2014 in Sierra Leone, one of the poorest countries in the world [20]. When the Ebola outbreak started in 2014, the country was still recovering from a long civil war that crippled the country between 1991 and 2002 [20]. A large majority of 80% of Sierra Leoneans have access to radio, but only 13% have access to newspapers [21]. Of the total population, 38% can surf the internet through computers or mobile phones, with up to 65% in urban areas [22]. The first survey was conducted in August (*n* = 1413) just 3 months into the outbreak and around the time it peaked in the Eastern Province. The second and third surveys were conducted about a month before (October, *n* = 2086) and a month after (December, *n* = 3540) the general peak of the outbreak with high transmission occurring in the Northern Province and Western Area. The sampling methods of the KAP surveys have been described in more detail elsewhere [7, 15]. In short, multistage cluster sampling was used, for which the 2004 Sierra Leone Population and Housing Census List of Enumeration Areas served as the sampling frame. In the first KAP survey, 9 out of the 14 districts of Sierra Leone were included, focusing mainly on the districts where Ebola cases were reported at that time. As the virus spread throughout the country, all districts were included in surveys 2 and 3. Within the randomly selected enumeration areas, the data collector started in the centre of the enumeration area where a pen was dropped. In the direction of the tip of the pen, households were approached based on a predetermined skip interval. In every household, two people were interviewed: the head of the household and either a younger person (age 15–24 years) or a woman age 25 or above. All three surveys aimed to produce national and regional-level estimates at a 95% confidence interval, within a 2.5% margin of error for national estimates and a 3.5% margin of error for regional estimates.

The level of risk perception of respondents was measured in the KAP surveys by asking ‘*what level of risk do you think you have in getting Ebola in the next 6 month*s?’ to which respondents could answer ‘no risk, small, moderate or great’. Answers were dichotomised into ‘no risk perception’ and ‘some level of risk perception’.

Exposure to different information sources was ascertained by the question ‘*Through what means/ways did you learn about Ebola?*’ This was an open question, where data collectors selected one or more pre-coded options that most closely matched the responses provided. Additional response options were incorporated into the second and third surveys. Mutually exclusive categories were subsequently made by grouping similar information sources into five categories, taking local media landscape into account: electronic media (radio and television), print media (newspapers, brochures and other print materials), new media (mobile phones, text messages, internet), government (house visits by health workers, governments campaigns) and community (religious and traditional leaders, megaphone public announcements, community meetings, friends and relatives). Information sources were also categorized by how many sources someone was exposed to: 0–1 source, 2, 3 and 4–5 sources. Because sample sizes for 0 and 5 sources were low (*n* = 24 and *n* = 159 respectively), they were combined with 1 and 4 sources.

Ebola-specific knowledge was ascertained in the KAP surveys through 2 open-ended and 5 closed-ended questions (see Table S1 in supplementary file). Two scores were created from this: a knowledge score with a maximum score of 8 points in surveys 1–3 and a misconceptions score, with a maximum score of 12. The scores were dichotomised based on the means [15].

Three Ebola-specific behaviours that are important to transmission control were included in this analysis as outcomes: washing hands with soap and water, avoiding physical contact with people suspected to have Ebola, and avoiding burials that involve contact with the corpse. These behaviours were ascertained through an open question: ‘*In what ways have you changed your behaviour or took actions to avoid being infected?’*

Analyses were adjusted for the following covariates: sex (male, female), age (15–20, 21–35, 36–49, 50+ years), education (no education, primary education, secondary and above), religion (Islam, Christianity) and region (Northern Province, Eastern Province, Southern Province, Western Area).

### Statistical analyses

In an outbreak situation that varies in intensity in different regions at different time points, it is likely that there is a high correlation between time and region. This violates the statistical assumption of independent observations. To account for this, data were analysed using multilevel modelling. In total across the 3 surveys, 167 sampling clusters were used on the first level, after which data were analysed on the individual level. Original survey weights were not applied, as the samples were collected proportionally to the district size in the population and the response rate was 98%. Within the multilevel models, associations were estimated with odds ratios (ORs) and their 95% confidence intervals (CIs). Four variables had some missing data; age (*n* = 28), education (*n* = 42), religion (*n* = 24) and sex (*n* = 12). Respondents with missing data (*n* = 104, 2% of the sample) were excluded from the multilevel analyses to allow for complete-case analysis.

Mediation analyses were carried out using the mediated effect model [19] to estimate if knowledge, misconceptions and Ebola-specific behaviours had a mediating effect on risk perception. From the adjusted models, the β coefficients for A and B were obtained (see figure S1 in supplementary file), after which they were multiplied. The χ2 distribution within 1 degree of freedom was used to determine statistical significance of the mediated effect. The analyses were carried out in Stata 15 and α was set to 0.05 for statistical significance.

Ethical permission for the surveys was granted by the Sierra Leone Research and Ethics Review Committee and approved by the Ethical Review Board at Karolinska Institutet in Stockholm, Sweden (dnr 2018/1276–31).

## Results

Pooling data from the three surveys resulted in a total sample size of 7039 respondents. Descriptive statistics of the demographics (Table 1) show that across the four regions in Sierra Leone, around one third of respondents were between 21 and 35 years old. Females comprised 51% of the total sample. Secondary education was attained by 52% of respondents, and the highest education attainment was reported from the Western Area (where the capital city Freetown is located), with 68% of the respondents attaining secondary education or higher levels. Among the four geographic regions, the Northern Province had the largest share of non-educated respondents (38%). Islam was the most common religious affiliation across the sample (65%) and in all regions. Between 50 and 69% of respondents expressed some level of risk perception during the first survey in the four regions. This decreased during the second survey for all regions apart from the Northern Province.

**Table 1:** Demographic characteristics and risk perception of the pooled sample

	Western Area N (%)	Northern Province N (%)	Eastern Province N (%)	Southern Province N (%)	Total N (%)
**Age (years)**					
15–20	388 (22)	507 (22)	382 (24)	319 (24)	1596 (23)
21–35	652 (37)	745 (32)	546 (34)	461 (34)	2404 (34)
36–49	443 (25)	534 (23)	370 (23)	301 (23)	1648 (24)
50+	274 (16)	513 (22)	307 (19)	257 (19)	1351 (19)
**Sex**					
Female	941 (53)	1112 (48)	838 (52)	702 (52)	3593 (51)
Male	821 (47)	1200 (52)	768 (48)	645 (48)	3434 (49)
**Education**					
No education	325 (19)	877 (38)	573 (36)	332 (25)	2107 (30)
Primary education	235 (13)	355 (15)	370 (23)	265 (20)	1225 (18)
Secondary or higher	1194 (68)	1065 (46)	644 (41)	742 (55)	3665 (52)
**Religion**					
Islam	919 (52)	1878 (81)	1003 (62)	778 (58)	4578 (65)
Christianity	837 (48)	430 (19)	603 (38)	567 (42)	2437 (35)
**Perceived some risk**					
Survey 1 (Aug 2014)	235 (55%)	302 (69%)	135 (50%)	166 (60%)	838 (59%)
Survey 2 (Oct 2014)	196 (38%)	427 (68%)	124 (30%)	156 (31%)	903 (43%)
Survey 3 (Dec 2014)	295 (36%)	635 (51%)	408 (44%)	189 (34%)	1527 (43%)

Missing data

Age: 40 (0.6%), Sex: 12 (0.2%), Education: 42 (0.6%), Religion: 24 (0.3%)

### Demographic determinants of risk perception

Having secondary school education or higher was positively associated with expressing Ebola risk perception (adjusted OR [AOR] 1.35, 95% CI 1.14–1.61) compared to having no education. Residing in the Northern Province was strongly associated with expressing Ebola risk perception compared to residing in the Western Area (AOR 2.70, 95% CI 1.87–3.89). Risk perception was significantly lower in the second survey in October 2014 (AOR 0.40, 95% CI 0.25–0.64) and third survey in December 2014 (AOR 0.37, 95% CI 0.25–0.56) compared to the baseline survey in August 2014 (Table 2).

**Table 2:** Association between sociodemographic covariates, level of outbreak and risk perception of getting Ebola

	Some Risk Perception N (%)	Crude OR (95% CI)	***P***-value	Adjusted OR^**a**^ (95% CI)	P-value
**Age (years)**					
15–20	698 (44)	1.0 (Reference)		1.0 (Reference)	
21–35	1128 (47)	1.15 (1.01–1.31)	0.039	1.24 (1.08–1.41)	0.002
36–49	803 (49)	1.20 (1.04–1.39)	0.015	1.27 (1.10–1.48)	0.002
50+	615 (46)	0.99 (0.83–1.17)	0.900	1.08 (0.90–1.31)	0.397
**Sex**					
Female	1599 (46)	1.0 (Reference)		1.0 (Reference)	
Male	1667 (49)	1.22 (1.09–1.36)	0.001	1.17 (1.05–1.32)	0.006
**Education**					
No education	919 (44)	1.0 (Reference)		1.0 (Reference)	
Primary	516 (42)	1.12 (0.92–1.36)	0.251	1.14 (0.93–1.40)	0.220
Secondary +	1811 (49)	1.42 (1.21–1.66)	0.000	1.35 (1.14–1.61)	0.001
**Religion**					
Islam	2138 (47)	1.0 (Reference)		1.0 (Reference)	
Christianity	1122 (46)	1.18 (1.03–1.36)	0.019	1.15 (0.99–1.33)	0.059
**Region**					
Western	726 (41)	1.0 (Reference)		1.0 (Reference)	
Northern	1364 (59)	2.28 (1.57–3.30)	0.000	2.70 (1.87–3.89)	0.000
Eastern	667 (41)	1.00 (0.64–1.55)	0.999	1.15 (0.75–1.79)	0.519
Southern	511 (38)	0.75 (0.44–1.26)	0.268	0.79 (0.48–1.31)	0.363
**Survey 1,2,3**^**b**^					
Survey 1 (Early in the outbreak)	838 (59)	1.0 (Reference)		1.0 (Reference)	
Survey 2 (Just before the peak of the outbreak)	903 (43)	0.44 (0.26–0.74)	0.002	0.40 (0.25–0.64)	0.000
Survey 3 (Just after the peak of the outbreak)	1527 (43)	0.46 (0.30–0.70)	0.000	0.37 (0.25–0.56)	0.000

^**a**^Adjusted for: age, sex, education, religion, region, KAP survey, all information sources

^**b**^Survey 1: August 2014; Survey 2: October 2014; Survey 3: December 2014

### Information sources and risk perception

In the crude and adjusted models, two information sources had a positive association with expressing risk perception (Table 3): new media (AOR 1.53, 95% CI 1.21–1.93) and community sources (AOR 1.28, 95% CI 1.07–1.53). Print media had a borderline positive association with expressing risk perception (AOR 1.24, 95% CI 1.00–1.54). Compared to people who were exposed to none or just one of the information sources, exposure to any three sources was associated with increased risk perception (AOR 1.50, 95% CI 1.12–2.00). Exposure to four or five sources had an even stronger association (AOR 1.73, 95% CI 1.18–2.52).

**Table 3:** Association between exposure to information sources and risk perception of getting Ebola

	No (%) Respondents	Crude OR (95% CI)	***P***-value	Adjusted OR^**a**^ (95% CI)	***P***-value
**Electronic media**					
Yes	6615 (94)	1.10 (0.78–1.55)	0.592	1.02 (0.72–1.44)	0.927
No	424 (6)	1.0 (Reference)		1.0 (Reference)	
**Print Media**					
Yes	679 (10)	1.49 (1.20–1.85)	0.000	1.24 (1.00–1.54)	0.046
No	6360 (90)	1.0 (Reference)		1.0 (Reference)	
**New Media**					
Yes	1012 (14)	1.69 (1.33–2.13)	0.000	1.53 (1.21–1.93)	0.000
No	6027 (86)	1.0 (Reference)		1.0 (Reference)	
**Government**					
Yes	3206 (46)	1.07 (0.84–1.36)	0.584	1.01 (0.80–1.27)	0.958
No	3833 (54)	1.0 (Reference)		1.0 (Reference	
**Community**					
Yes	4133 (59)	1.34 (1.11–1.60)	0.002	1.28 (1.07–1.53)	0.008
No	2906 (41)	1.0 (Reference)		1.0 (Reference	
**No. Exposures**^**b**^					
0–1	1916 (27)	1.0 (Reference)		1.0 (Reference)	
2	2450 (35)	1.18 (0.96–1.46)	0.118	1.18 (0.95–1.46)	0.132
3	1998 (28)	1.56 (1.17–2.07)	0.003	1.50 (1.12–2.00)	0.006
4–5	675 (10)	1.83 (1.25–2.67)	0.002	1.72 (1.17–2.51)	0.006

^**a**^Adjusted for: age, sex, education, religion, region, KAP survey, all other information sources

^**b**^Adjusted model includes: age, sex, education, religion, region, KAP survey

### Risk perception and Ebola-specific knowledge and behaviours

In the fully adjusted models, Ebola-specific knowledge was positively associated with expressing Ebola risk perception (AOR 1.42, 95% CI 1.15–1.75), see Table 4. Having misconceptions on the other hand was negatively associated with risk perception (AOR 0.67, 95% CI 0.55–0.82). In terms of behaviours, hand washing had a positive association with risk perception (AOR 1.40, 95% CI 1.13–1.74) and avoiding burials was negatively associated with risk perception (AOR 0.77, 95% CI 0.62–0.96). There was no association between avoiding physical contact with Ebola-suspects and risk perception (AOR 1.08, 95% CI 0.91–1.29).

**Table 4:** Association between knowledge, misconceptions, behaviours and risk perception of getting Ebola

	Crude OR (95% CI)	***P***-value	Adjusted OR^**a**^ (95% CI)	***P***-value	Adjusted OR^**b**^ (95% CI)	***P***-value
**Knowledge**						
No	1.0 (Reference)		1.0 (Reference)		1.0 (Reference)	
Yes	1.50 (1.21–1.86)	0.000	1.46 (1.17–1.81)	0.001	1.42 (1.15–1.75)	0.001
**Misconceptions**						
No	1.0 (Reference)		1.0 (Reference)		1.0 (Reference)	
Yes	0.66 (0.54–0.80)	0.000	0.69 (0.56–0.83)	0.000	0.67 (0.55–0.82)	0.000
**Hand washing**						
No	1.0 (Reference)		1.0 (Reference)		1.0 (Reference)	
Yes	1.45 (1.17–1.81)	0.001	1.45 (1.16–1.81)	0.000	1.40 (1.13–1.74)	0.002
**Avoid physical contact**						
No	1.0 (Reference)		1.0 (Reference)		1.0 (Reference)	
Yes	1.17 (0.97–1.40)	0.094	1.13 (0.95–1.35)	0.178	1.08 (0.91–1.29)	0.380
**Avoid burials**						
No	1.0 (Reference)		1.0 (Reference)		1.0 (Reference)	
Yes	0.81 (0.66–1.01)	0.059	0.80 (0.64–0.99)	0.043	0.77 (0.62–0.96)	0.023

^**a**^Adjusted for: region, sex, age, religion, education, KAP survey

^**b**^Adjusted for: region, sex, age, religion, education, KAP survey and all information sources

### Mediation analysis

Knowledge and misconceptions played a mediating role in the association between all information sources (apart from electronic media) and risk perception, see Table 5. Hand washing only mediated the association between government and community sources and risk perception. Whereas there was no direct association between avoiding physical contact with suspected Ebola patients and risk perception (Table 4), this behaviour mediated the association between all information sources apart from electronic media and risk perception.

**Table 5:** Mediation analyses

Category	Beta A (SE)	Beta B (SE)	Beta AB (SE)	***P***-value
**Knowledge to risk perception of getting Ebola**				
Electronic media	0.656 (0.115)	0.350 (0.108)	0.230 (0.082)	0.185
Print media	0.216 (0.108)	0.350 (0.108)	0.076 (0.044)	0.015
New media	0.344 (0.117)	0.350 (0.108)	0.120 (0.055)	0.050
Government	0.388 (0.092)	0.350 (0.108)	0.136 (0.053)	0.041
Community	0.307 (0.086)	0.350 (0.108)	0.107 (0.045)	0.016
2 sources	0.197 (0.096)	0.342 (0.107)	0.068 (0.039)	0.006
3 sources	0.603 (0.115)	0.342 (0.107)	0.207 (0.076)	0.157
4–5 sources	1.237 (0.186)	0.342 (0.107)	0.424 (0.147)	0.466
**Misconceptions to risk perception of getting Ebola**				
Electronic media	0.082 (0.150)	−0.398 (0.101)	−0.033 (0.060)	0.094
Print media	0.056 (0.128)	−0.398 (0.101)	−0.022 (0.051)	0.049
New media	0.147 (0.119)	−0.398 (0.101)	− 0.059 (0.050)	0.042
Government	0.218 (0.099)	−0.398 (0.101)	− 0.087 (0.045)	0.025
Community	0.174 (0.101)	−0.398 (0.101)	− 0.069 (0.044)	0.021
2 sources	−0.019 (0.106)	− 0.401 (0.100)	0.008 (0.043)	0.019
3 sources	0.341 (0.129)	−0.401 (0.100)	− 0.137 (0.062)	0.107
4–5 sources	0.430 (0.189)	−0.401 (0.100)	− 0.172 (0.087)	0.251
**Hand washing to risk perception of getting Ebola**				
Electronic media	0.357 (0.156)	0.338 (0.109)	0.121 (0.066)	0.096
Print media	0.241 (0.157)	0.338 (0.109)	0.082 (0.059)	0.066
New media	0.313 (0.142)	0.338 (0.109)	0.106 (0.059)	0.064
Government	0.282 (0.104)	0.338 (0.109)	0.096 (0.047)	0.020
Community	0.328 (0.080)	0.338 (0.109)	0.111 (0.045)	0.015
2 sources	0.329 (0.089)	0.337 (0.109)	0.111 (0.047)	0.020
3 sources	0.638 (0.124)	0.337 (0.109)	0.215 (0.081)	0.178
4–5 sources	0.915 (0.227)	0.337 (0.109)	0.308 (0.126)	0.385
**Avoiding physical contact to risk perception of getting Ebola**				
Electronic media	0.534 (0.169)	0.078 (0.088)	0.041 (0.049)	0.071
Print media	0.411 (0.106)	0.078 (0.088)	0.032 (0.037)	0.018
New media	0.459 (0.108)	0.078 (0.088)	0.036 (0.041)	0.033
Government	0.369 (0.080)	0.078 (0.088)	0.029 (0.033)	0.008
Community	0.255 (0.081)	0.078 (0.088)	0.020 (0.023)	0.000
2 sources	0.191 (0.103)	0.079 (0.088)	0.015 (0.019)	0.000
3 sources	0.666 (0.120)	0.079 (0.088)	0.042 (0.060)	0.138
4–5 sources	1.132 (0.165)	0.079 (0.088)	0.089 (0.101)	0.381
**Avoiding unsafe burial to risk perception of getting Ebola**				
Electronic media	0.565 (0.209)	−0.255 (0.111)	−0.144 (0.082)	0.178
Print media	0.609 (0.133)	−0.255 (0.111)	−0.156 (0.076)	0.143
New media	0.297 (0.103)	−0.255 (0.111)	−0.076 (0.042)	0.009
Government	0.225 (0.106)	−0.255 (0.111)	−0.057 (0.037)	0.003
Community	0.432 (0.097)	−0.255 (0.111)	−0.110 (0.054)	0.040
2 sources	0.182 (0.133)	−0.249 (0.110)	− 0.045 (0.039)	0.004
3 sources	0.630 (0.158)	−0.249 (0.110)	− 0.157 (0.080)	0.167
4–5 sources	1.178 (0.220)	−0.249 (0.110)	− 0.293 (0.141)	0.434

## Discussion

This study shows that exposure to print media, new media or community-based sources of information was associated with increased risk perception of getting Ebola in the next 6 months from the time of being interviewed. Ebola-specific knowledge and hand washing were positively associated with risk perception, Ebola-specific misconceptions and avoiding unsafe burials were negatively associated with risk perception. Mediation analyses revealed that knowledge, misconceptions and Ebola-specific behaviours mediated the associations between exposure to various information sources and risk perception.

Whereas risk perception is deemed to be a key determinant to elicit behaviour change, previous studies have shown ambiguous results when testing the role of risk perception in changing the public’s behaviour [23–25]. Our findings regarding various behaviours and risk perception similarly go in various directions. Brewer’s [19] observation that many studies use cross-sectional surveys to interpret the influence of risk perception on behaviours might explain this inconsistency. As Brewer points out in the ‘risk reappraisal hypothesis’, over time, and especially in an ever-evolving situation such as the Ebola outbreak, adopted behaviours might in turn influence the level of perceived risk [19]. The observed positive association between hand washing and risk perception can therefore be interpreted in various ways; those who feel at risk are more inclined to wash their hands. Or, those who wash their hands do so for a reason; they felt at risk. Similarly, the negative association between avoiding burials and risk perception can be interpreted in this direction: people who avoided unsafe traditional burials did not perceive themselves to be at risk of getting Ebola.

Ideally, temporal data is used to test this hypothesis [19]. Our data, while collected at three progressive time periods that aligned with different stages of the outbreak, is considered cross-sectional data. However, our results show that over time, overall risk perception decreased compared to the first survey in August 2014. This is in line with the actual risk of transmission over time and might indicate that by adopting protective behaviours, the perception of risk was lowered over the course of the outbreak - supporting the risk reappraisal hypothesis.

Risk perception has been described to have two components: a subjective component based on feelings and an analytical component based on available facts [26]. Whereas in theory the analytical component should make calculations based on actual risk, it has been shown that people are relatively insensitive to understanding probability. The subjective component will often take over, whereby feelings determine the level of perceived risk [26]. The finding that risk perception decreased over time, might therefore point to successes in risk communication to match the public’s subjective risk to the actual risk of transmission. However, as many other interventions were implemented concurrently, we cannot discern the size of the effect of any risk communication intervention using an observational design. Furthermore, social learning may have occurred intrinsically without external interventions. For instance, when people observed fewer cases of Ebola in their communities, districts, and country, they might have felt less at risk of getting Ebola. Therefore, intrinsic social learning may also contribute in explaining why risk perceptions declined sharply between the first survey (administered before the peak of the outbreak) and third survey (administered after the peak).

Exposure to community-based information sources through community leaders, community meetings and friends and relatives was associated with expressing Ebola risk perception. Together, these community-based sources form a group of people who are highly trusted and respected [27]. Whereas the initial response to the outbreak was criticized for prioritizing top-down risk communication messaging instead of focussing on community engagement, local leaders were actively engaged later in the outbreak response [28]. Community sources and new media are comparable in that they both facilitate active interaction between the messenger and the recipient of that message [27]. As opposed to top-down messaging, this form of two-way communication allows recipients to ask questions, express concerns and discuss solutions [29]. It is plausible that by potentially getting a better understanding of the disease and the outbreak through these interactions, the perception of risk might have been enhanced.

Having Ebola-specific knowledge was also positively associated with risk perception. It is plausible that people with enhanced risk perception sought more knowledge, even though with this study design it is equally possible that people with increased knowledge may have elevated risk perception. However, an online survey in the USA found that higher knowledge was associated with decreased perceptions of risk about Ebola [30]. In another online survey in Germany, there was no association between Ebola-specific knowledge and being worried about Ebola [5]. The closeness and actual immediate risk in Sierra Leone, and the accuracy of risk perceptions might have played a role in this [16]. It is also possible that a combination of the directions of the association occurred. However, with the available data we cannot discern this. Finally, having misconceptions about Ebola was negatively associated with perceiving some level of risk. This is an important finding, as misconceptions can also be associated with risk behaviour [15]. A survey from the North Kivu and Ituri Ebola outbreak in the Democratic Republic of the Congo shows that belief in misinformation was associated with a decreased likelihood of adopting protective behaviours [31].

### Strengths and limitations

A major strength of this study was the relatively large sample sizes of the three KAP surveys. The surveys captured population-level data during an ongoing large-scale outbreak at several timepoints across all geographic regions of Sierra Leone. The sampling method and the high response rates should mitigate the risk of selection bias. The KAP survey instruments were not validated because of the ongoing emergency and the need of gathering data rapidly. However, the survey instruments were pilot-tested and KAP surveys have shown to be a useful tool in several outbreaks [7, 8, 31, 32]. Risk perception was measured using a generic unidimensional Likert-item in the three KAP surveys that our analyses are based on. Models and theories of risk perception and behavioural change assert that risk perception is a complex phenomenon that is driven by the multiplicity of affective responses to the hazard, subjective appraisals of its likelihood, and perceptions of its consequences [33]. Given the multidimensionality of risk perception, future Ebola (or similar epidemic) risk perception assessments should consider including multiple items that reflect the underlying domains of risk perception such as affect, probability, and consequence [16].

Exposure to information sources was dichotomised into exposed and unexposed. We did not have information on the content of the messaging, the framing, the tone or the amount of actual coverage. These factors can all potentially play a role in amplifying or attenuating the perceived risk – especially in a fluid and chaotic outbreak context [34]. Moreover, we carried out mediation analyses with cross-sectional data, and therefore cannot establish if the assumed exposure to information sources happened before the assumed mediators (knowledge, misconceptions, and behaviour) and outcome (risk perception). However, it is plausible that the public in Sierra Leone first learned about the existence of an outbreak through at least one of the information sources we included in this study.

We attempted to account for the high correlation between time and region by applying multilevel modelling. Whereas we adjusted for several demographic factors, unmeasured, residual confounding could have had an influence on the outcomes. The three surveys covered all 4 regions in Sierra Leone, but contrary to survey 2 and 3, survey 1 only covered 9 out of 14 districts. To mitigate this limitation, the multilevel approach accounted for the effects of geographic clusters – which were more granular than districts.

## Conclusion

Our results provide novel insights into the complex relationship between risk perception and human behaviour during an unprecedented Ebola outbreak. Exposure to community-level information sources was positively associated with perceiving some Ebola risk. More people perceived themselves to be at risk before the peak of the outbreak compared to after the peak. Individuals who said they took protective actions against Ebola perceived themselves to be at decreased risk of getting Ebola. These findings reinforce the importance of effective risk communication, especially in the early stages of an outbreak, to help the public understand their risk and take appropriate actions to reduce their acquisition risk. Community-level information sources may help to align the public’s perceived risk with their actual epidemiological risk through continuous exchange of information that help to improve knowledge, reduce misconceptions, and facilitate uptake of protective behaviours. As part of global health security efforts, increased investments are needed for community-level engagements that allow for two-way communication during health emergencies.

## Supplementary information

**Additional file 1 : Table S1.** Composite variables for Ebola-specific knowledge and misconceptions. **Figure S1.** Mediation analysis among information exposure, knowledge, behaviour and risk perception, Sierra Leone, 2014.

## Data Availability

The datasets used and/or analysed during the current study are available from the corresponding author on reasonable request.

## Abbreviations

AOR: Adjusted odds ratio
CI: Confidence interval
KAP: Knowledge, attitudes, practices
OR: Odds ratio
SE: Standard error
