# Supplementary material for: Risk perception during the 2014–2015 Ebola outbreak in Sierra Leone
Source: BMC Public Health. 2020 Oct 12;20:1539. doi: 10.1186/s12889-020-09648-8 (PMC7549333; doi:10.1186/s12889-020-09648-8)
Supplement: Supplementary file 1 — Additional file 1 : Table S1. Composite variables for Ebola-specific knowledge and misconceptions. Figure S1. Mediation analysis among information exposure, knowledge, behaviour and risk perception, Sierra Leone, 2014. [file 12889_2020_9648_MOESM1_ESM.docx]

**Supplementary material**

**Risk perception during the 2014-2015 Ebola outbreak in Sierra Leone**

| **Knowledge Questions** | **Correct answers** | **Incorrect Answers** |
| --- | --- | --- |
| **Open Question** | | |
| What causes Ebola? | 1. Virus  2. Bats/monkeys/chimpanzees | 1. God or higher power  2. Witchcraft  3. Evildoing/sin  4. Curse |
| What happens if someone suspected of Ebola goes to the hospital? | They will take care of him/her (rehydrate, give medicine/food, monitor status) | 1. They won’t be able to do anything, they may die there  2. They will definitely cure Ebola  3. They will find a way to kill the patient |
| **Closed Questions** | | |
| Can I prevent myself from getting Ebola by avoiding funeral/burial rituals that require handling the body of someone who died from Ebola? | Yes | No |
| If a person has Ebola, does he/she have a higher chance of survival if he/she goes immediately to a health facility? | Yes | No |
| If a person with Ebola goes immediately to a health facility will he/she reduce the chance of spreading it to family or people living with? | Yes | No |
| Do you believe that traditional healers can treat Ebola successfully? | No | Yes |
| Do you believe that spiritual healers can treat Ebola successfully? | No | Yes |
| Maximum score | KAP 1-3: 8 | KAP 1-3: 12 |
| Cut Off | KAP 1-3: 0-6/7-8 | KAP 1-3: 0/>=1 |

**Table S1.** Composite variables for Ebola-specific knowledge and misconceptions

**Figure S1.** Mediation analysis among information exposure, knowledge, behaviour and risk perception, Sierra Leone, 2014.
